# A Pedunculated Neoplasm of the Thigh

**Published:** 2017-07-24

**Authors:** Erica Y. Xue, Jerette J. Schultz, Ramazi Datiashvili

**Affiliations:** Division of Plastic and Reconstructive Surgery, Department of Surgery, Rutgers/New Jersey Medical School, Newark

**Keywords:** thigh lesion, fibrolipoma, pedunculated, lipoma, neoplasm

**Figure 1:**
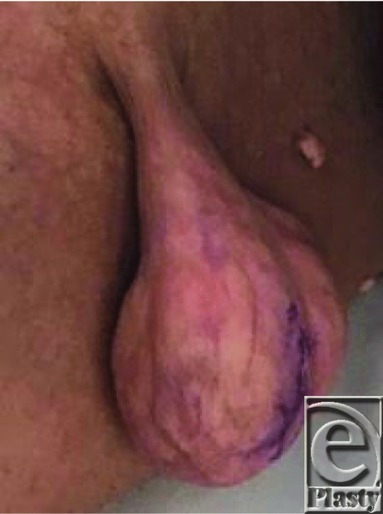
Preoperative view of the pedunculated upper thigh mass.

## DESCRIPTION

A 47-year-old man presented to the plastic surgery clinic with a pedunculated lesion of the right medial thigh. The lesion had been present for the majority of the patient's life and had gradually enlarged to the point where it was interfering with wearing pants.

## QUESTIONS

1. **What is the differential diagnosis for a single, flesh-toned, pedunculated tumor of the thigh?**
2. **What is a fibrolipoma?**
3. **What is the presentation of other reported pedunculated fibrolipomas?**
4. **How are fibrolipomas managed?**

### DISCUSSION

The differential diagnosis for a pedunculated thigh mass includes acrochordon, solitary nevus lipomatosus cutaneous superficialis (NLCS), and lipoma. Acrochordons, also known as skin tags, are 2- to 6-mm pedunculated papules or nodules found in the collar, groin, and axillary areas; they are common, have a genetic tendency, and may be seen in association with metabolic syndrome.^1^ NLCS is a benign, hamartomatous condition characterized by the presence of mature ectopic adipocytes in the dermis. NLCS not only presents most commonly in the classical form as multiple nodules, papules, or plaques but can also appear as a single, dome-shaped papule in the solitary form.^2^ Lipomas are the most common benign soft-tissue tumors; their true prevalence is difficult to establish, since they are diagnosed clinically and often left untreated. There are many subtypes of lipomas, including fibrolipoma, angiolipoma, myxoid lipoma, spindle cell lipoma, pleomorphic lipomas, and intramuscular lipomas.

The final pathology report of the excised tumor on our patient was fibrolipoma, a microscopic variant of lipoma characterized by fibrous connective tissue intermixed with fat lobules.^3^ Fibrolipomas are commonly found in the oral mucosa, although there are also reported cases of fibrolipoma in various other anatomic areas. They are postulated to arise from the lipoblastomatosis, a benign infiltrative neoplasm composed of immature fat cells, connective tissue septa, and loose myxoid matrix; the adipocytes and fibrous tissue mature into a fibrolipoma.^4^

Fibrolipomas are generally not pedunculated. There are only a few pedunculated fibrolipomas reported in the literature that vary in size and location. The smallest reported pedunculated fibrolipoma originated from the buccal mucosa and measured 1.9 × 1.4 × 0.5 cm.^4^ The largest reported pedunculated fibrolipoma was located in the facial and neck region and involved the cervical sheath, submandibular gland, and buccal fat pad; it measured 45 × 40 × 46 cm and weighed 6 kg.^5^ Two other reported cases of pedunculated fibrolipoma were in the vulva and plantar surface of the foot.^6^^,^^7^ While the reported lesions were histologically benign, they were frequently associated with complications such as skin ulceration from repeated friction and inflammation, as well as dysphagia in oroesophageal fibrolipomas.

Definitive treatment of lipomas is usually surgical excision. Complete resection is necessary to prevent recurrence. Newer technology, such as diode laser surgery, has been successfully used to excise a small fibrolipoma of the lip.^8^ Our patient's pedunculated thigh fibrolipoma was excised in an elliptical fashion; the mass was 4.2 × 4.2 × 1.8 cm and weighed 38 g.

While lipomas are the most common benign soft-tissue tumors, pedunculated fibrolipoma is a rare case. Fibrolipomas are a histologic subset of lipoma, composed of mature adipocytes intermixed with fibrous connective tissue. The few reported fibrolipomas have varied in dimensions and anatomic location. Although not life-threatening, pedunculated fibrolipomas may ulcerate or cause other functional inconvenience; surgical excision is the definitive treatment.
